# 25-Hydroxyvitamin D levels among 2-year-old children: findings from the Japan environment and Children’s study (JECS)

**DOI:** 10.1186/s12887-021-03005-3

**Published:** 2021-12-02

**Authors:** Limin Yang, Miori Sato, Mayako Saito-Abe, Makoto Irahara, Minaho Nishizato, Hatoko Sasaki, Mizuho Konishi, Kazue Ishitsuka, Hidetoshi Mezawa, Kiwako Yamamoto-Hanada, Yukihiro Ohya, for the Japan Environment and Children’s Study (JECS) Group), Michihiro Kamijima, Shin Yamazaki, Yukihiro Ohya, Nobuo Yaegashi, Koichi Hashimoto, Chisato Mori, Shuichi Ito, Zentaro Yamagata, Hidekuni Inadera, Takeo Nakayama, Hiroyasu Iso, Masayuki Shima, Youichi Kurozawa, Narufumi Suganuma, Koichi Kusuhara, Takahiko Katoh, Reiko Kishi

**Affiliations:** 1 Division of Allergy, Department of Medical Subspecialties, Medical Support Center for Japan Environment and Children’s Study (JECS), Allergy Center, National Center for Child Health and Development, 2-10-1, Okura, Setagayaku, Tokyo, 157-8535 Japan; 2 Medical Support Center for the Japan Environment and Children’s Study, National Research Institute for Child Health and Development, Tokyo, Japan

**Keywords:** 25-Hydroxyvitamin D, Vitamin D, Deficiency, Insufficiency, Children, Cohort

## Abstract

**Background:**

The study aim was to obtain epidemiological data on vitamin D levels for the pediatric population in Japan. We assessed the prevalence of vitamin D deficiency and insufficiency in 2-year-old Japanese children using data from a large ongoing birth cohort study.

**Methods:**

Data for analysis was obtained from the Japan Environment and Children’s Study (JECS) and a Sub-Cohort Study (SCS) of JECS. We evaluated the children’s serum 25(OH) D levels by 5th, 10th, 25th, 50th, 75th, 90th, and 95th percentiles, and the rates of vitamin D deficiency or insufficiency. We also presented a weighted prevalence rate for vitamin D deficiency or insufficiency among all children in JECS.

**Results:**

After excluding children with missing 25(OH)D2 or 25(OH)D3 data, we analyzed 4655 remaining children, of whom 24.7% (95% CI, 23.5–26.0%) had vitamin D deficiency (< 20 ng/mL), and 51.3% (95% CI, 49.8–52.7%) were at risk of vitamin D insufficiency (20–30 ng/mL). The estimated prevalence of vitamin D deficiency and insufficiency among all children in JECS were 25.4% (95% CI, 24.1–26.7%) and 50.9% (95% CI, 49.4–52.4%). Vitamin D deficiency was found in 22.9% of boys and 26.5% of girls. Median serum 25(OH) D concentrations were lower among participants measured during winter and spring than among those measured in summer and autumn. The highest rate of vitamin D deficiency was observed in Hokkaido, the northernmost prefecture of Japan.

**Conclusion:**

We analyzed data on serum 25(OH) D levels from a birth cohort study and found that vitamin D deficiency and insufficiency are very common among 2-year-old Japanese children. Sex, season, and latitude affect serum 25(OH) D concentrations.

**Supplementary Information:**

The online version contains supplementary material available at 10.1186/s12887-021-03005-3.

## Background

Vitamin D in its hormonal active form, 1,25-dihydroxyvitamin D, functions in regulating calcium levels and phosphate homoeostasis, and is important for bone growth and metabolism [1]. Vitamin D deficiency contributes to two metabolic bone diseases: rickets in children and osteomalacia in adults [1–4]. In addition, vitamin D helps regulate the immune system and cell proliferation and differentiation. Vitamin D deficiency is associated with a wide range of chronic diseases and conditions, including infections, asthma, adverse pregnancy outcomes, cancer, autoimmune disease, cardiovascular diseases, and multiple sclerosis [5–12]. Diet, supplement use, age, geographic latitude, culture, lifestyle, avoiding sunlight exposure, skin pigmentation, and individual differences in vitamin metabolism are well-known risk factors for low vitamin D levels [1]. A recent review of vitamin D status implied vitamin D deficiency was widespread, regardless of a country’s economic level and latitude [1]. Around 1 billion people in the world are suspected to have vitamin D deficiency or insufficiency [13]. In Europe, it might affect more than 50% of the population, depending on the season [14].

Serum 25-hydroxyvitamin D (25(OH)D) is a major circulating metabolite of vitamin D, which has a long half-life in the body, relative stability, and plentiful concentration in the blood. Thus, serum 25(OH) D concentration is the best indicator for recent vitamin D input among those without kidney disease [15–17]. Published studies suggest that serum 25(OH)D3 levels of ≥30 ng/mL are optimal for bone metabolism; levels between 20 and 29 ng/mL indicate insufficiency; and levels < 20 ng/mL suggests vitamin D deficiency [18]·. However, consensus on the optimal serum concentration of 25(OH) D has not been demonstrated yet [19].

Few studies have evaluated the prevalence of vitamin D deficiency in children in Japan. One study testing serum 25(OH) D levels in 574 3-year-old Japanese children, living at latitude 35°N, found 29.6% children had insufficient vitamin D (< 20 ng/mL) [20]. Another study found that 17% of 132 Japanese infants had serum 25(OH) D levels < 20 ng/mL [21].

The study aim was to obtain epidemiological data on vitamin D levels in the pediatric population in Japan using an accurate and reliable measurement approach. Accordingly, we examined serum 25(OH) D levels to assess the prevalence of vitamin D deficiency and insufficiency in 2-year-old Japanese children using data from a large ongoing birth cohort study.

## Method

### Study design

Data for analysis was obtained from the Japan Environment and Children’s Study (JECS) and a Sub-Cohort Study (SCS) of JECS. The JECS is a large-scale birth cohort study launched by the Ministry of the Environment, Japan in 2011. The cohort study includes 15 Regional Centres (Hokkaido, Miyagi, Fukushima, Chiba, Kanagawa, Koshin, Toyama, Aichi, Kyoto, Osaka, Hyogo, Tottori, Kochi, Fukuoka, and South Kyushu/Okinawa). Participants were pregnant woman and their partners who were recruited at first trimester of pregnancy when the Maternal and Child Health Handbook was issued or at clinic when woman got a diagnosis of pregnancy. The main aim of the JECS was to evaluate associations between environmental factors and children’s health and development. Over 100,000 pregnant women were recruited between January 2011 and March 2014. Children in the cohort will be followed up until they reach 13 years of age. The Main Study uses information from all the recruited participants. JECS protocols are described in detail elsewhere [22–24].

In addition to the Main Study, JECS is conducting an SCS with 5017 participants randomly selected from all the participants in the Main Study [25]. The participants of SCS are children in the Main Study who were born after 1 April 2013, and met all of the following requirements: 1) have a response to all the questionnaire surveys conducted during the period from the first trimester of pregnancy to 6 months after delivery, and data of medical records collected during the same period should be available, 2) data of biospecimens (except for umbilical cord blood) collected at the first trimester, second/third trimester, and delivery should be available [26].

Data on blood test was derived from the SCS. Blood samples of 4695 2-year-old children were collected and analyzed by contract lab in JECS. Lidocaine and prilocaine cream were used to relief pain associated with venipuncture [27] .The total amount of blood collected at 2-year-olds was 4 ml. Liquid chromatography-tandem mass spectrometry (LC-MS/MS) (LSI Medience Corporation; Japan) was used to measure serum 25(OH)D3 and 25(OH)D2 concentrations. The system was calibrated with‘6PLUS1 Multilevel Serum Calibrator Set 25-OH-Vitamin D3/D2’. The sample testing was validated through Accuracy-Based Vitamin D Survey (ABVD). The intra- assay CV for 25(OH)D3 was L: 9.9% (17.2 ng/mL) and H:9.1% (44.2 ng/mL). The inter-assay CV for 25(OH)D3 was L: 4.7% (15.7 ng/mL) and H:5.2% (39.3 ng/mL).

Missing on 25(OH)D2 or 25(OH)D3 were excluded from analysis. Because there were only 40 missing cases (< 5%), excluding these cases will not affect results significantly. Data used for analysis are from the jecs-ta-20,190,930-qsn dataset, and the jecs-ta-20,190,930-mdv dataset, released by the Program Office in October 2019.

The SCS was performed based on the guidelines specified in the Declaration of Helsinki. All procedures involving human participants were approved by the Japan Ministry of the Environment’s Institutional Review Board on Epidemiological Studies (No. 100406001) and the ethics committees of all participating institutions. Written consent to the SCS protocol is signed separately from that of the Main Study and obtained from all participating mothers and fathers [26].

### Cut-points for serum 25(OH)D

Values for 25(OH)D2 or 25(OH)D3 concentrations below 4 ng/mL were truncated as 4 ng/mL. Total serum 25(OH) D levels was defined as the sum of serum 25(OH)D3 and 25(OH)D2 concentrations. To clarify rates of vitamin D deficiency and insufficiency in this population, we considered those with serum 25(OH) D levels of < 20 ng/mL at risk of deficiency, 20–29 ng/mL at risk of insufficiency, and ≥ 30 ng/mL to have sufficient levels, according to the assessment criteria published by the Japanese Society for Bone and Mineral Research [28].

### Other variables

Data on the participating children’s sex and region of residence were collected from medical record transcripts during the participating mothers’ pregnancy and at the children’s births. With respect to seasons when participants’ blood was drawn, we considered March–May for spring, June–August for summer, September–November for autumn, and December–February for winter.

### Statistical analysis

We examined rates of vitamin D deficiency (less than 20 ng/mL) or insufficiency (20–29 ng/mL) by sex, season, and Regional Center. Categorical variables were summarized using proportions (%). Serum 25(OH) D level was also used as a continuous variable to describe the distribution according to the 5th, 10th, 25th, 50th, 75th, 90th, and 95th percentiles, and to evaluate relationships with sex, season, and latitude. The difference in 25(OH) D levels between boys and girls was evaluated using the Wilcoxon test. The Kruskal–Wallis test was used to compare medians of serum 25(OH) D concentrations between the four season groups. Wilcoxon tests were performed for multiple comparisons after the Kruskal–Wallis test, and *P* values were adjusted using the Bonferroni correction. The strength and direction of association between latitude and serum 25(OH) D concentrations was assessed using the Spearman correlation coefficient.

To assess vitamin D status among all children in JECS, we also presented a weighted prevalence rate for 25(OH) D deficiency or insufficiency. The weights were the inverse of the predicted probability of participants by fitting a logistic model adjusting for maternal age, parental degrees, family income, birthweight, and child’s sex. Descriptive analysis, models, and bootstrap processes were performed using R version 3.6.1 software (Institute for Statistics and Mathematics, Vienna, Austria; www.r-project.org).

## Results

After excluding those with missing data for serum 25(OH)D2 or D3 concentrations, 4655 cases were left for analysis. Rates of vitamin D deficiency or insufficiency are list in Tables 1. The 5th, 10th, 25th, 50th, 75th, 90th, and 95th percentiles of serum 25(OH)-D levels are listed in Additional file 2. The median serum 25(OH) D concentration was 24.7 ng/mL in SCS. We found 24.7% (95% CI, 23.5–26.0%) of children in SCS had a level below 20 ng/mL, and 51.3% (95% CI, 49.8–52.7%) were at risk of vitamin D insufficiency (20–30 ng/mL). A total of 22.9% (95% CI, 21.2–24.6%) of boys and 26.5% (95%CI, 24.7–28.4%) of girls had 25(OH) D levels less than 20 ng/mL, whereas 26.5% (95% CI, 24.7–28.3%) of boys and 21.6% (95% CI, 19.9–23.3%) of girls had serum 25(OH) D levels greater than 30 ng/mL.

**Table 1:** Prevalence rates of vitamin D deficiency or insufficiency among 2 years old children in SCS

	ALL (*N* = 4655)	Boys (*N* = 2364)	Girls (*N* = 2291)
25(OH)D	Prevalence (%) (95% CI)	Prevalence (%) (95% CI)	Prevalence (%) (95% CI)
< 20 ng/mL	24.7 (23.5–26.0)	22.9 (21.2–24.6)	26.5 (24.7–28.4)
> = 20 and < 30 ng/mL	51.3 (49.8–52.7)	50.6 (48.6–52.7)	51.9 (49.8–54.0)
> = 30 ng/mL	24.1 (22.8–25.3)	26.5 (24.7–28.3)	21.6 (19.9–23.3)

*CI* confidence interval; *25(OH) D* 25-hydroxyvitamin D; *SCS* Sub-Cohort Study

**Fig. 1:**
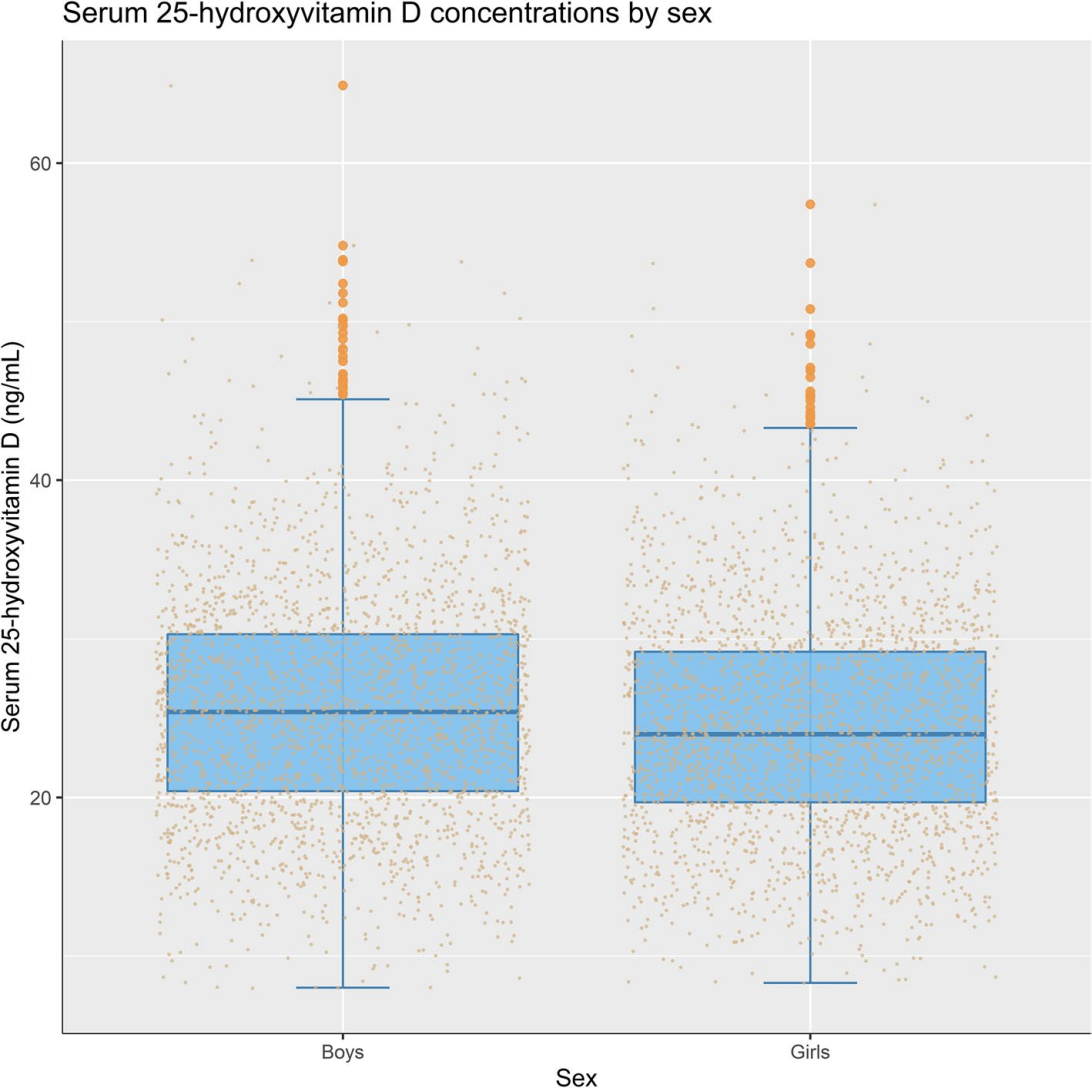
Serum 25-hydroxyvitamin D levels among 2-year-old children in SCS by sex. *P*_Wilcoxon test_ < 0.001. SCS: Sub-Cohort Study

Figure 1 shows serum 25(OH) D status by sex. The median 25(OH) D concentration was 25.4 ng/mL in boys, significantly higher than the 24.0 ng/mL in girls (*P*_Wilcoxon test_ < 0.001).

**Fig. 2:**
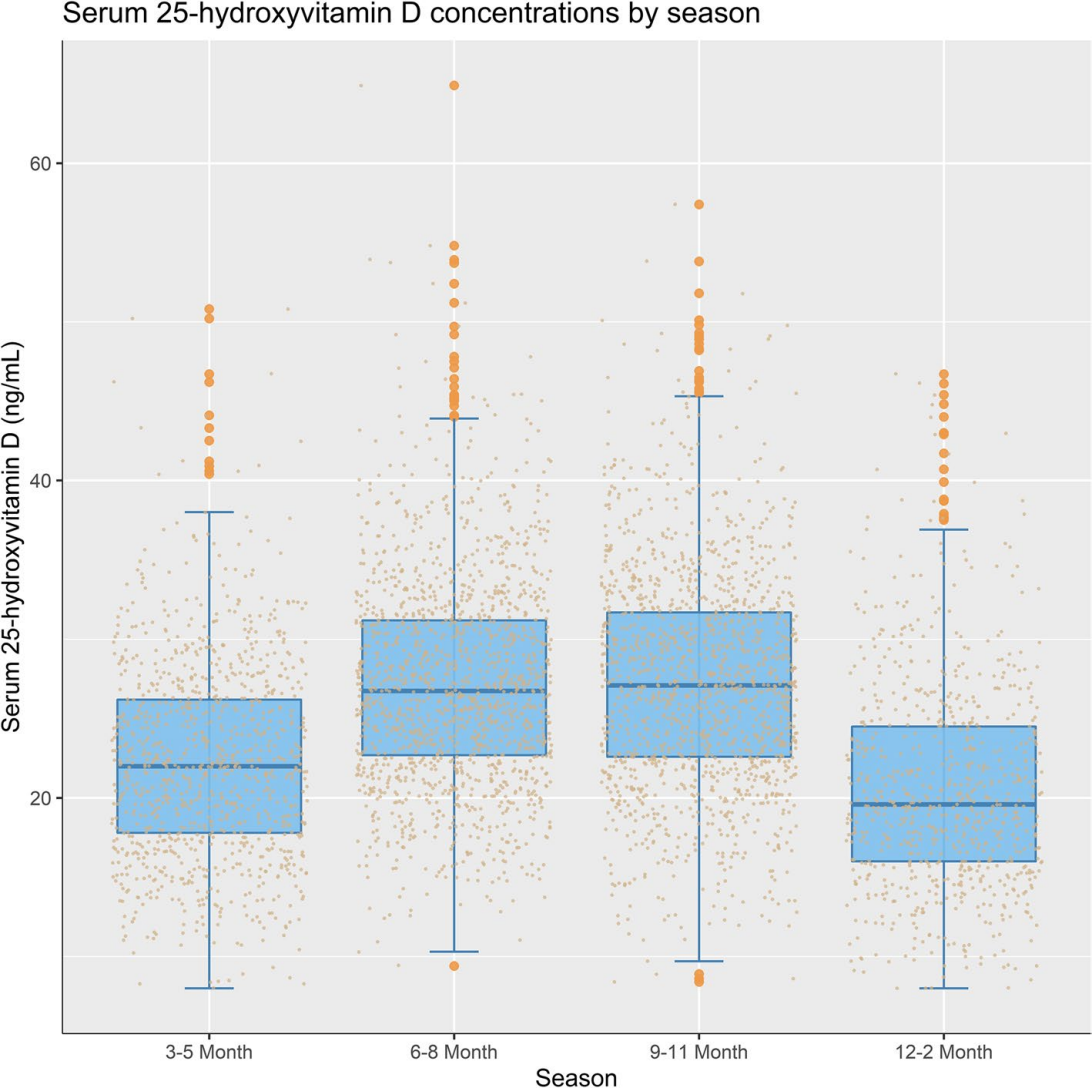
Serum 25-hydroxyvitamin D levels among 2-year-old children in SCS by season. *P*_Kruskal–Wallis test_ < 0.001. All the pairwise Wilcoxon tests showed significant between-group differences in distribution (*P* < 0.0001), except for spring vs autumn. SCS: Sub-Cohort Study

Figure 2 shows the distribution of serum 25(OH) D concentrations by season. The distribution was different between the four season groups (*P*_Kruskal–Wallis test_ < 0.001). The median serum 25(OH) D concentrations were lower among participants measured during winter and spring than among those measured in summer and autumn. All the pairwise Wilcoxon tests showed significant between-group differences in distribution (*P* < 0.0001), except for spring vs autumn.

**Fig. 3:**
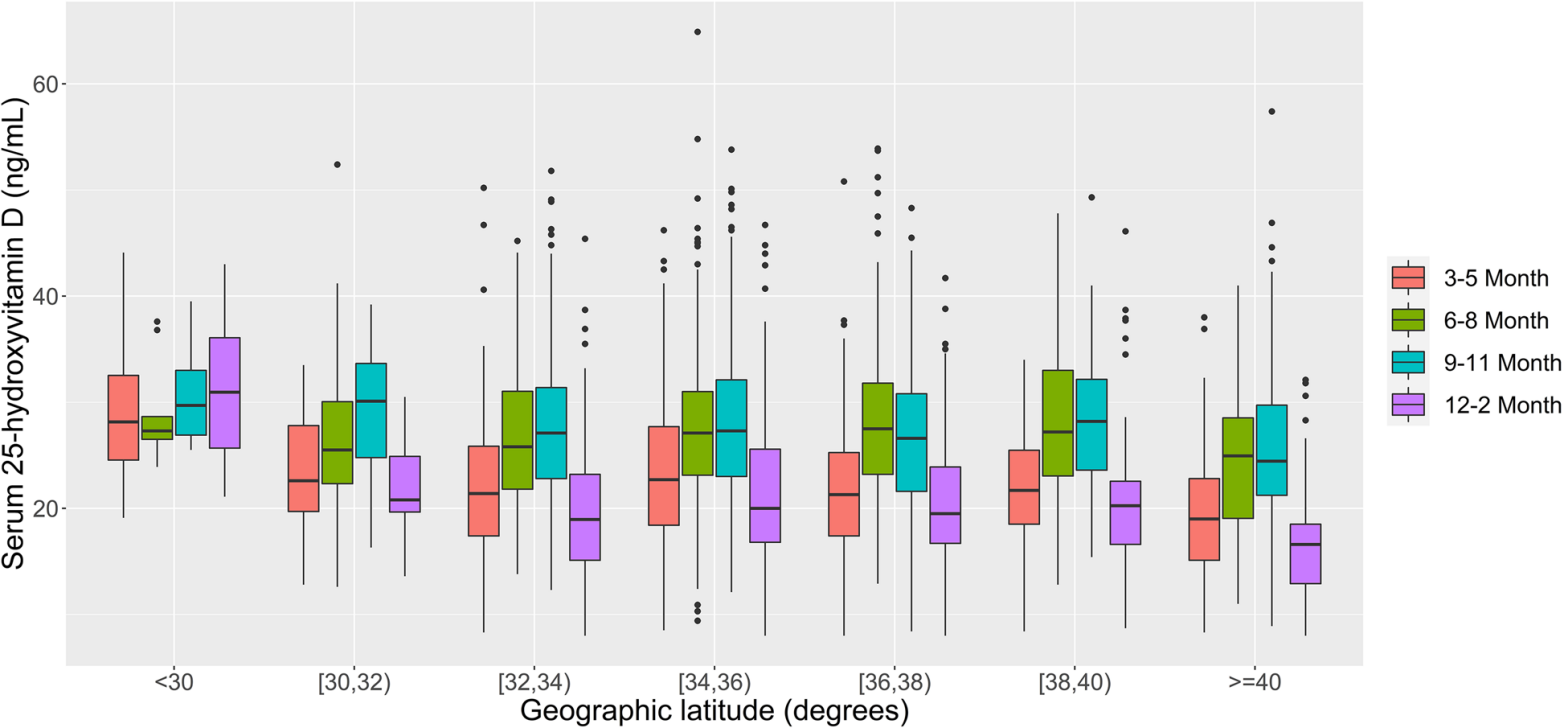
Serum 25-hydroxyvitamin D levels among 2-year-old children in SCS by latitude and season. SCS: Sub-Cohort Study

Figure 3 shows the serum 25(OH) D concentrations by latitude and season. We found a very weak but significant relationship between latitude and serum 25(OH) D concentrations (Spearman’s rank correlation rho = − 0.04, *P* = 0.004). Higher rates of vitamin D deficiency were found in areas north of 40°N, including Sapporo, the Asahikawa part of Kitami, Oketo, Kunneppu, Tsubetsu, and Bihoro, which are located in Hokkaido Prefecture; whereas lower rates were found in areas south of 30°N, explaining data from Okinawa, the southernmost prefecture of Japan. In winter, 80% of the children living in Hokkaido areas had deficient serum 25(OH) D levels (Additional file 1).

Estimated rates of vitamin D deficiency or insufficiency among 2 years old children in JECS are showed in Table 2. The estimated proportion of serum 25(OH) D levels less than 20 ng/mL and 20 to < 30 ng/mL among all children in JECS were 25.4% (95% CI, 24.1–26.7%) and 50.9% (95% CI, 49.4–52.4%). Back-weighted rates were similar to observed rates from the SCS.

**Table 2:** Estimated prevalence rates of vitamin D deficiency or insufficiency among 2 years old children in JECS

	ALL	Boys	Girls
25(OH)D	Population prevalence^#^ (%) (95% CI)	Population prevalence^#^ (%) (95% CI)	Population prevalence^#^ (%)(95% CI)
< 20 ng/mL	25.4 (24.1–26.7)	23.5 (21.7–25.3)	27.4 (25.5–29.3)
> = 20 and < 30 ng/mL	50.9 (49.4–52.4)	50.4 (48.3–52.5)	51.4 (49.2–53.5)
> = 30 ng/mL	23.7 (22.5–25.0)	26.1(24.3–27.9)	21.3 (19.5–23.0)

*25(OH) D* 25-Hydroxyvitamin D;*CI* confidence interval; *JECS* Japan Environment and Children’s Study

^#^The estimated prevalence rate was a weighted rate. The weights were the inverse of the predicted probability of participants by fitting a logistic model adjusting for maternal age, parental degrees, family income, birthweight, and child’s sex

## Discussion

This is the first effort to report serum 25(OH) D levels for 2-year-old Japanese children with a large sample. Here, we present descriptive statistics, using data from the JECS and SCS of JECS, which show approximately 20% of children in JECS had serum 25(OH) D levels less than 20 ng/mL, and approximately 70% had levels below 30 ng/mL.

High prevalence rates of vitamin D insufficiency or deficiency among the pediatric population have been observed in many countries. In U.K. population, (mainly white 7–11 yr olds), 29% were vitamin D deficient (< 20 ng/mL) and 75% had concentrations of total 25(OH) D less than 30 ng/mL [29]. Study based on a nationally representative sample of US children aged 1 to 21 years in the National Health and Nutrition Examination Survey 2001–2004 found that 9% of children had 25(OH) D levels less than 15 ng/mL, and 61% of the children had 25(OH) D levels between 15 and 29 ng/mL [30]. A recent study in Finland found almost 20% of the children (6–8 years) had serum 25(OH) D concentrations below 20 ng/mL, and about 70% of the children had serum 25(OH) D concentrations below 30 ng/mL [31]. Prevalence of vitamin D deficiency (≤20 ng/mL) among healthy infants and toddlers (380 primary care US children aged 8–24 months) was 12.1, and 40.0% had levels below 30 ng/mL [32]. A study in Japan mentioned above estimated an insufficient vitamin D (< 20 ng/mL) prevalence of 29.6% in 3-year-old Japanese children [20]. In 2012, Zhu et al. investigated the vitamin D status of children aged 1 month to 16 years in Huangzhou, China. They reported 22% of children aged 2–5 years lacked vitamin D(< 20 ng/mL) [33]. The variations in the prevalence of vitamin D insufficiency and deficiency could be explained by differences in study design, measurement methods, participant age, season of measurement, and resident latitude.

Our study indicates that girls had lower average 25(OH) D than boys, which is similar to findings from other countries. This may reflect differences in time spent playing outdoors rather than biological differences in vitamin D metabolism between boys and girls.

Our study also found regional differences in vitamin D levels among Japanese children. UV exposure may partly explain this geographical difference. People living in high latitudes are prone to vitamin D insufficiencies, especially in winter [34, 35]. The highest vitamin D deficiency rate was found in Hokkaido Prefecture, the northernmost regional Prefecture of Japan, which partly reflects the effects of sunlight exposure. Surprisingly, although located in southern areas, our finding also indicated that the springtime vitamin D deficiency rate was higher in the Fukuoka Regional Centre than in most other areas, even those located in more northern latitudes. We speculate that Aeolian dust blown during spring from semi-arid areas of the Asian continent may lead to the high rate of hypovitaminosis D observed in the Fukuoka Regional Centre. According to the Japan Meteorological Agency, the total days of observed Aeolian dust were nearly 1 week each during March 2013 and May 2014 in cities in Fukuoka, which is much higher than in other areas. Aeolian dust could decrease sunlight exposure, because people are inclined to stay indoors when Aeolian dust arrives [21].

The lack of access to vitamin D-fortified food and vitamin D supplements for children may partly explain the vitamin D deficiency or insufficiencies in Japanese children. Consumption of vitamin D-fortified food is minimal in Japan. In addition, there is no recommendation or guidelines for vitamin D supplementation for Japanese children [20].

One advantage of this study was the large number of participants from a large birth cohort study in Japan. Children in the JECS represented about 45% of live births in the catchment area of all Japanese Regional Centres, and their characteristics were similar to those of children in the Japanese Vital Statistics; thus results from the cohort can be generalized to the Japanese population. In addition, the study covers a wide geographical area from the northernmost prefecture (Hokkaido) to the southernmost prefecture (Okinawa), which allows us to explore the relationship with latitude and vitamin D status. We used LC-MS/MS to test serum 25(OH) D concentrations in current study. LC-MS/MS is widely accepted in clinical laboratories to differentiate between vitamin-D deficiency and sufficiency, because of its excellent analytical specificity and sensitivity [36–39].

The study has some limitations that should be considered when comparing with other studies. First, more than 60% of children in this cohort were tested during the summer and autumn, and time of year significantly affects serum 25(OH) D levels. As shown in Table 2, the prevalence rates of 25(OH) D less than 20 ng/mL in our study were 52.6% in winter vs 12.2% in summer; thus, the overall vitamin D deficiency rate might be underestimated here. In addition to time of year, when comparing serum 25(OH) D levels between studies, differences in latitude must be taken into account [31]. Second, values for serum 25(OH) D concentration that were lower than 4 ng/mL were truncated to 4 ng/mL in this analysis, which may cause serum 25(OH) D concentrations to be slightly overestimated. Finally, the lack of consensus on optimal vitamin D levels [19] and normal range for 25(OH) D concentration further complicates studies of serum 25(OH) D concentration. Moreover, serum 25(H) D concentration vary depending on the assays used for testing, which make defining vitamin deficiency with serum 25(OH) D concentration more difficult [40] [31]. Some studies suggest 30 ng/mL as the lower limit for sufficient serum 25(OH) D level [41]; others suggest 20 ng/mL [20, 42]. Only around 24% of children in our cohort had serum 25(OH) D levels > 30 ng/mL. Whether rates of vitamin D deficiency or insufficiency were overestimated by using the current cut-off value (> 30 ng/mL) also needs to be further assessed. Developing cut-off points for the definition of vitamin D status for young population is needed.

In conclusion, we analyzed data on serum 25(OH) D levels from a birth cohort study and found that vitamin D deficiency and insufficiency are very common among 2-year-old Japanese children. Sex, season, and latitude affect serum 25(OH) D concentrations. Further analysis of other factors that may affect vitamin D levels, such as supplementation and dietary intake, should be conducted.

## Supplementary Information

**Additional file 1:.**

**Additional file 2:.**

## Data Availability

The JECS data are not publicly available due to ethical restrictions and the legal framework of Japan. All inquiries about access to the data should be sent to the JECS Programme Office, National Institute for Environmental Studies (jecs-en@nies.go.jp).

## Abbreviations

25(OH)D: 25-hydroxyvitamin D
JECS: Japan Environment and Children’s Study (JECS)
CI: Confidence interval
SCS: Sub-Cohort Study
