# Supplementary material for: 25-Hydroxyvitamin D levels among 2-year-old children: findings from the Japan environment and Children’s study (JECS)
Source: BMC Pediatr. 2021 Dec 2;21:539. doi: 10.1186/s12887-021-03005-3 (PMC8638176; doi:10.1186/s12887-021-03005-3)
Supplement: Supplementary file 1 — Additional file 1:. [file 12887_2021_3005_MOESM1_ESM.docx]

**Additional file 1** Prevalence rates and 95% CI of vitamin D deficiency or insufficiency among 2 years old children in SCS by Regional Centres and seasons

|  |  | Spring | |  | Summer | |  | Autumn | |  | Winter | |  |
| --- | --- | --- | --- | --- | --- | --- | --- | --- | --- | --- | --- | --- | --- |
| Regional Centres | 25(OH)D | % | 95% CI |  | % | 95% CI |  | % | 95% CI |  | % | 95% CI |  |
| Hokkaido | <20 ng/mL | 56.2 | 45.3-66.5 |  | 27.7 | 19.8-37.1 |  | 21.1 | 14.2-29.9 |  | 80.0 | 64.9-89.9 |  |
|  | >=20 and <30 ng/mL | 39.3 | 29.3-50.3 |  | 52.7 | 43.1-62.1 |  | 54.4 | 44.8-63.7 |  | 13.3 | 5.5-27.5 |  |
| Miyagi | <20 ng/mL | 38.9 | 29.0-49.8 |  | 9.2 | 4.9-16.2 |  | 9.7 | 5.0-17.5 |  | 48.7 | 37.3-60.2 |  |
|  | >=20 and <30 ng/mL | 53.3 | 42.6-63.8 |  | 56.7 | 47.3-65.6 |  | 52.4 | 42.4-62.3 |  | 43.6 | 32.6-55.3 |  |
| Fukushima | <20 ng/mL | 37.6 | 29.2-46.7 |  | 10.4 | 6.6-15.8 |  | 17.1 | 11.9-23.7 |  | 55.6 | 44.1-66.5 |  |
|  | >=20 and <30 ng/mL | 52.8 | 43.7-61.7 |  | 57.5 | 50.2-64.5 |  | 52.9 | 45.2-60.6 |  | 37.0 | 26.8-48.5 |  |
| Chiba | <20 ng/mL | 32.7 | 21.0-46.8 |  | 3.3 | 0.9-10.0 |  | 12.1 | 6.5-21.0 |  | 43.5 | 29.2-58.8 |  |
|  | >=20 and <30 ng/mL | 43.6 | 30.6-57.6 |  | 58.2 | 47.4-68.3 |  | 47.3 | 36.8-57.9 |  | 45.7 | 31.2-60.8 |  |
| Kanagawa | <20 ng/mL | 30.1 | 20.2-42.1 |  | 8.0 | 3.6-16.4 |  | 6.7 | 2.7-14.5 |  | 43.5 | 29.2-58.8 |  |
|  | >=20 and <30 ng/mL | 56.2 | 44.1-67.6 |  | 54.0 | 43.0-64.6 |  | 58.9 | 48.0-69.0 |  | 50.0 | 36.1-63.9 |  |
| Koshin | <20 ng/mL | 26.9 | 17.8-38.4 |  | 6.9 | 3.1-14.2 |  | 11.8 | 6.5-20.0 |  | 33.3 | 22.3-46.4 |  |
|  | >=20 and <30 ng/mL | 60.3 | 48.5-71.0 |  | 54.5 | 44.3-64.3 |  | 48.0 | 38.1-58.1 |  | 52.4 | 39.5-65.0 |  |
| Toyama | <20 ng/mL | 43.1 | 29.6-57.7 |  | 10.0 | 4.5-20.1 |  | 19.4 | 12.4-28.9 |  | 53.8 | 37.4-69.6 |  |
|  | >=20 and <30 ng/mL | 54.9 | 40.5-68.6 |  | 51.4 | 39.3-63.4 |  | 53.1 | 42.8-63.1 |  | 38.5 | 23.8-55.3 |  |
| Aichi | <20 ng/mL | 37.1 | 25.4-50.3 |  | 9.8 | 4.6-18.8 |  | 10.4 | 4.9-20.0 |  | 65.0 | 48.3-78.9 |  |
|  | >=20 and <30 ng/mL | 53.2 | 40.2-65.8 |  | 63.4 | 52.0-73.6 |  | 54.5 | 42.8-65.8 |  | 20.0 | 9.6-36.1 |  |
| Kyoto | <20 ng/mL | 24.1 | 11.0-43.9 |  | 8.9 | 3.3-20.4 |  | 10.9 | 4.5-22.9 |  | 54.3 | 36.9-70.8 |  |
|  | >=20 and <30 ng/mL | 51.7 | 32.9-70.1 |  | 57.1 | 43.3-70.0 |  | 47.3 | 33.9-61.1 |  | 42.9 | 26.8-60.5 |  |
| Osaka | <20 ng/mL | 40.8 | 29.8-52.7 |  | 15.7 | 9.8-23.9 |  | 21.2 | 14.4-29.9 |  | 54.2 | 40.8-67.1 |  |
|  | >=20 and <30 ng/mL | 47.4 | 35.9-59.1 |  | 57.4 | 47.8-66.5 |  | 50.8 | 41.5-60.1 |  | 37.3 | 25.3-50.9 |  |
| Hyogo | <20 ng/mL | 32.5 | 19.1-49.2 |  | 10.4 | 5.4-18.7 |  | 9.2 | 3.8-19.7 |  | 54.8 | 38.8-69.8 |  |
|  | >=20 and <30 ng/mL | 55.0 | 38.7-70.4 |  | 54.2 | 43.7-64.3 |  | 52.3 | 39.6-64.7 |  | 33.3 | 20.0-49.6 |  |
| Tottori | <20 ng/mL | 43.3 | 26.0-62.3 |  | 13.6 | 5.7-28.0 |  | 11.9 | 4.5-26.4 |  | 46.2 | 27.1-66.3 |  |
|  | >=20 and <30 ng/mL | 43.3 | 26.0-62.3 |  | 59.1 | 43.3-73.3 |  | 52.4 | 36.6-67.7 |  | 42.3 | 24.0-62.8 |  |
| Kochi | <20 ng/mL | 33.3 | 23.3-45.0 |  | 11.7 | 6.3-20.4 |  | 5.4 | 2.2-11.8 |  | 54.3 | 39.2-68.8 |  |
|  | >=20 and <30 ng/mL | 43.3 | 26.0-62.3 |  | 50.0 | 40.1-59.9 |  | 53.6 | 43.9-63.0 |  | 39.1 | 25.5-54.6 |  |
| Fukuoka | <20 ng/mL | 46.3 | 35.4-57.6 |  | 20.7 | 14.0-29.4 |  | 15.3 | 9.1-24.3 |  | 58.2 | 45.5-69.9 |  |
|  | >=20 and <30 ng/mL | 51.2 | 40.0-62.3 |  | 60.3 | 50.8-69.2 |  | 54.1 | 43.7-64.1 |  | 35.8 | 24.7-48.5 |  |
| South Kyushu | <20 ng/mL | 28.8 | 17.5-43.3 |  | 12.6 | 6.8-21.9 |  | 9.2 | 4.3-17.8 |  | 47.8 | 33.1-62.9 |  |
|  | >=20 and <30 ng/mL | 55.8 | 41.4-69.3 |  | 55.2 | 44.2-65.7 |  | 58.6 | 47.6-68.9 |  | 39.1 | 25.5-54.6 |  |
| Total | <20 ng/mL | 37.7 | 34.7-40.8 |  | 12.2 | 10.6-14.0 |  | 13.4 | 11.7-15.3 |  | 52.6 | 48.9-56.2 |  |
|  | >=20 and <30 ng/mL | 51.6 | 48.5-54.7 |  | 56.1 | 53.6-58.7 |  | 52.8 | 50.2-55.4 |  | 38.5 | 35.0-42.0 |  |

CI: confidence interval; 25(OH)D: 25-hydroxyvitamin D; SCS: Sub-Cohort Study.
