# Supplementary material for: 25-Hydroxyvitamin D levels among 2-year-old children: findings from the Japan environment and Children’s study (JECS)
Source: BMC Pediatr. 2021 Dec 2;21:539. doi: 10.1186/s12887-021-03005-3 (PMC8638176; doi:10.1186/s12887-021-03005-3)
Supplement: Supplementary file 2 — Additional file 2:. [file 12887_2021_3005_MOESM2_ESM.docx]

**Additional file 2** Serum 25-hydroxyvitamin D status among 2 years old children in SCS

|  |  | Percentile | | | | | | |  |
| --- | --- | --- | --- | --- | --- | --- | --- | --- | --- |
| Regional Centres | N | 5th | 10th | 25th | 50th | 75th | 90th | 95th | 95% CI of median# |
| Hokkaido | 360 | 11.4 | 13.7 | 17.0 | 22.2 | 27.4 | 32.2 | 36.1 | 21.4-22.9 |
| Miyagi | 391 | 14.1 | 16.6 | 20.2 | 24.5 | 29.7 | 34.6 | 37.5 | 23.3-25.5 |
| Fukushima | 569 | 13.5 | 15.6 | 20.1 | 24.7 | 29.8 | 34.9 | 38.4 | 24.0-25.4 |
| Chiba | 283 | 14.4 | 16.3 | 21.9 | 26.4 | 30.7 | 35.4 | 40.9 | 25.0-27.7 |
| Kanagawa | 296 | 15.5 | 17.7 | 21.2 | 25.6 | 30.2 | 34.1 | 37.6 | 24.4-26.7 |
| Koshin | 344 | 15.8 | 17.7 | 21.2 | 25.8 | 30.5 | 34.9 | 37.8 | 24.8-26.9 |
| Toyama | 258 | 14.1 | 16.1 | 19.8 | 24.2 | 29.2 | 32.9 | 37.4 | 23.1-25.3 |
| Aichi | 261 | 14.4 | 16.6 | 20.0 | 24.7 | 29.2 | 33.4 | 36.0 | 23.8-25.7 |
| Kyoto | 175 | 15.0 | 17.6 | 21.3 | 26.6 | 30.7 | 34.5 | 38.9 | 24.9-27.3 |
| Osaka | 368 | 14.1 | 15.8 | 19.4 | 23.5 | 28.6 | 33.3 | 36.3 | 22.9-24.5 |
| Hyogo | 243 | 14.8 | 16.7 | 20.3 | 25.8 | 30.6 | 33.8 | 37.3 | 24.6-26.7 |
| Tottori | 142 | 15.0 | 15.9 | 20.0 | 24.9 | 29.2 | 35.1 | 36.8 | 23.5-26.4 |
| Kochi | 330 | 15.4 | 17.7 | 20.9 | 25.6 | 30.8 | 35.4 | 37.4 | 24.8-26.5 |
| Fukuoka | 363 | 12.5 | 14.9 | 18.9 | 23.3 | 27.6 | 32.1 | 36.0 | 22.2-24.1 |
| South Kyushu | 272 | 14.3 | 16.8 | 20.7 | 25.4 | 30.0 | 34.8 | 37.4 | 24.4-26.9 |
| Total | 4655 | 14.0 | 16.0 | 20.0 | 24.7 | 29.7 | 34.4 | 37.6 | 24.4-25,0 |

CI: confidence interval; SCS: Sub-Cohort Study
